# Comparison between the *HLA-B*^∗^*58 : 01* Allele and Single-Nucleotide Polymorphisms in Chromosome 6 for Prediction of Allopurinol-Induced Severe Cutaneous Adverse Reactions

**DOI:** 10.1155/2017/2738784

**Published:** 2017-12-17

**Authors:** Niwat Saksit, Nontaya Nakkam, Parinya Konyoung, Usanee Khunarkornsiri, Wongwiwat Tassaneeyakul, Pansu Chumworathayi, Sirimas Kanjanawart, Chonlaphat Sukasem, Alisara Sangviroon, Oranuch Pattanacheewapull, Wichittra Tassaneeyakul

**Affiliations:** ^1^Department of Pharmacology, Faculty of Medicine, Khon Kaen University, Khon Kaen, Thailand; ^2^School of Pharmaceutical Sciences, University of Phayao, Phayao, Thailand; ^3^Pharmacy Unit, Udon Thani Hospital, Udon Thani, Thailand; ^4^Faculty of Pharmaceutical Sciences, Khon Kaen University, Khon Kaen, Thailand; ^5^Pharmacy Unit, Faculty of Medicine, Khon Kaen University, Khon Kaen, Thailand; ^6^Department of Pathology, Division of Pharmacogenomics and Personalized Medicine, Faculty of Medicine Ramathibodi Hospital, Mahidol University, Bangkok, Thailand; ^7^Laboratory for Pharmacogenomics, Somdech Phra Debaratana Medical Center (SDMC), Faculty of Medicine Ramathibodi Hospital, Mahidol University, Bangkok, Thailand; ^8^Pharmacy Unit, Police General Hospital, Bangkok, Thailand; ^9^Pharmacy Department, Khon Kaen Hospital, Khon Kaen, Thailand

## Abstract

Severe cutaneous adverse drug reactions (SCARs) are life-threatening reactions. The strong association between the *HLA-B*^∗^*58 : 01* allele and allopurinol-induced SCARs is well recognized. Screening for *HLA-B*^∗^*58 : 01* allele before prescribing allopurinol in some populations has been recommended. Several single-nucleotide polymorphisms (SNPs) in chromosome 6 have been found to be tightly linked with the *HLA* allele, and these SNPs have been proposed as surrogate markers of the *HLA-B*^∗^*58 : 01* allele. This study aimed to evaluate the association between three SNPs in chromosome 6 and allopurinol-induced SCARs in a Thai population. The linkage disequilibrium between the *HLA-B*^∗^*58 : 01* allele and these SNPs was also evaluated. Results showed that three SNPs including rs9263726, rs2734583, and rs3099844 were significantly associated with allopurinol-induced SCARs but with a lower degree of association when compared with the *HLA-B*^∗^*58 : 01* allele. The sensitivity, specificity, PPV, and NPV of these SNPs were comparable to those of the *HLA-B*^∗^*58 : 01* allele. Although detection of the SNP is simpler and less expensive compared with that of the *HLA-B*^∗^*58 : 01* allele, these SNPs were not perfectly linked with the *HLA-B*^∗^*58 : 01* allele. Screening using these SNPs as surrogate markers of the *HLA-B*^∗^*58 : 01* allele to avoid SCARs prior to allopurinol administration needs caution because of their imperfect linkage with the *HLA-B*^∗^*58 : 01* allele.

## 1. Introduction

Allopurinol, a uric acid-lowering agent, is one of the most common culprit drugs for severe cutaneous adverse drug reactions (SCARs). These reactions range from Stevens-Johnson syndrome (SJS) and toxic epidermal necrolysis (TEN) to drug reaction with eosinophilia and systemic symptoms (DRESS). Although the incidence of allopurinol-induced SCARs is rare, they are life-threatening reactions. Data from systematic reviews shows that although the prevalence of gout in Asian population is lower than that in Caucasian populations, the hypersensitivity caused by allopurinol reported in Asians was about 73% of the reported cases [1]. In Taiwan, the annual incidence rates for allopurinol hypersensitivity were 4.68 per 1000 new users, 2.02 per 1000 new users for related hospitalization, and 0.39 per 1000 new users for related mortality [2]. The mortality rate of allopurinol hypersensitivity in Taiwan was about 8.3% [2]. Similarly, the incidence rate of allopurinol-induced SCARs in Thailand reported from the biggest hospital in Thailand was about 2.13 per 1000 new users [3]. Compared with that of other drug-induced SCARs, the mortality rate of allopurinol-induced SCARs observed in a Thai population is the highest up to 11% [4].

Although allopurinol-induced SCARs are considered as idiosyncratic reactions, current studies have identified several risk factors of such fatal reactions that include both genetic and nongenetic factors [4–6]. For genetic factors, the specific allele of the human leukocyte antigen (HLA), namely, the *HLA-B*^∗^*58 : 01* allele, is the first genetic marker that was found to be strongly associated with allopurinol-induced SCARs in a Taiwanese population [7]. This association has been confirmed in Thai [4, 8, 9] and Han Chinese [10, 11] populations. Unlike the *HLA-B*^∗^*15 : 02* allele which is specific to Chinese and Southeast Asian population, the associations between the *HLA-B*^∗^*58 : 01* allele and allopurinol-induced SCARs were also demonstrated in Japanese [12, 13], Korean [14], and Caucasian populations [15]. The strength of association ranges from an odds ratio (OR) of 39 to 696 [16], and the sensitivity and specificity of the *HLA-B*^∗^*58 : 01* allele for the prediction of allopurinol-induced SCARs were 93% (95% CI: 85–97%) and 89% (95% CI, 87–91%) across Asian and Caucasian populations [16]. To date, the American College of Rheumatology recommends the testing for the *HLA-B*^∗^*58 : 01* allele in certain ethnicities with a high frequency of this allele and showing an elevated risk for allopurinol-induced SCARs in *HLA-B*^∗^*58 : 01* allele carriers such as Han Chinese, Thai, and Korean populations [17].

Due to the highly polymorphic nature of the *HLA* gene, a specific method is required in order to determine a specific *HLA* allele, particularly the *HLA-B* alleles in which exon 2 and 3 regions exhibit the highest variability. Several molecular techniques including specific sequencing primers (SSP) PCR, sequence-specific oligonucleotide (SSO) probes, and sequencing-based typing (SBT) have been demonstrated to be specific methods for the determination of *HLA* genotype; however, these techniques are quite expensive, time-consuming, and not commonly available in hospital laboratories. A recent genome-wide association study in a Japanese population has discovered a number of single-nucleotide polymorphisms (SNPs) in chromosome 6 that were strongly associated with allopurinol-induced SCARs [13]. These SNPs included rs2734583 in the *HLA-B-associated transcript 1* (*BAT1*) gene, rs3099844 in the *HLA complex P5* (*HCP5*) gene, and rs9263726 in the *psoriasis susceptibility 1 candidate 1* (*PSORS1C1*) gene. Due to the absolute linkage disequilibrium between rs9263726 and the *HLA-B*^∗^*58 : 01* allele found in 27 Japanese patients who suffered from allopurinol-induced SCARs, the rs9263726 has been proposed as a surrogate marker for allopurinol-induced SJS/TEN [18]. Similarly, an absolute linkage disequilibrium between the rs9263726 and allopurinol-induced SCARs has also been recently reported in 17 Eastern Chinese patients [19]. Ethnic specificity of the associations between *HLA* alleles and drug-induced SCARs is well recognized [20]. Whether these SNPs are good surrogates of allopurinol-induced SCARs in other ethnic societies or not needs to be evaluated. The present study aimed to evaluate the degree of relationship between the three selected SNPs in chromosome 6 including rs9263726, rs2734583, and rs3099844 and allopurinol-induced SCARs in a Thai population that has a relatively high frequency of the *HLA-B*^∗^*58 : 01* allele. In addition, the sensitivity and specificity for these selected SNPs for the prediction of allopurinol-induced SCARs and their linkage disequilibrium with the *HLA-B*^∗^*58 : 01* allele are characterized in the present study.

## 2. Materials and Methods

### 2.1. Study Population Assessment and Enrollment

A total of 96 allopurinol-induced SCARs patients including 23 DRESS and 73 SJS/TEN patients were recruited for the study. These patients had been diagnosed with allopurinol-induced SCARs within the first 3 months of allopurinol exposure. The phenotype of SCARs in an individual patient was scored and assessed by the ALDEN [21] or the RegiSCAR algorithms [22] whereas the assessment of causative drugs was performed using Naranjo's algorithm [23]. All of the SCARs patients who represented at least a probable score were recruited for the study.

For the control cohort comparison, 193 patients were recruited from patients who had used allopurinol for more than 6 months without any evidence of cutaneous reactions. Written informed consent was obtained from each patient. The study protocol within the hospital network of Khon Kaen University was approved by the Khon Kaen Ethics Committee for Human Research, Khon Kaen University, Thailand (HE510837). The study protocol was also approved by each hospital, where IRB function was available.

### 2.2. Genomic DNA Preparation

Leukocytes were separated from peripheral blood by centrifugation at 3500 rpm for 15 min or buccal swab. Genomic DNA (gDNA) was isolated from leukocytes using a QIAamp DNA Blood mini kit (QIAGEN GmbH, Hilden, Germany). The quantity and quality of gDNA were checked by a Nano Drop machine (Thermo Scientific NanoDrop 2000c) and kept at −20°C until used.

### 2.3. Detection of the *HLA-B*^∗^*58 : 01* Allele

The *HLA-B*^∗^*58 : 01* allele was detected using a commercial PG5801 DNA detection kit (Pharmigene Inc., Taipei, Taiwan) as described previously [8]. The *HLA-B* genotypes of patients who had the *HLA-B*^∗^*58 : 01* allele were confirmed using the PCR sequence-specific oligonucleotide probe method as has been described in a previous study [24].

### 2.4. Detection of rs9263726, rs2734583, and rs3099844 SNPs

The rs9263726 in the *PSORS1C1* gene was detected using the polymerase chain reaction-restriction fragment length polymorphism (PCR-RFLP) assay as previously described [18]. The PCR conditions were set at 94°C for 5 min followed by 35 cycles of amplification at 94°C for 30 sec, 60°C for 45 sec, and 72°C for 45 sec and final extension of 72°C for 7 min. The 260 bp length PCR products were digested with Fok I endonuclease (New England Biolabs, Beverly, MA, USA), and the DNA fragments were detected by agarose gel electrophoresis.

The rs2734583 in the *BAT1* gene (assay ID: C__26778946_20) and the rs3099844 in the *HCP5* gene (assay ID: C__27455402_10) were detected by TaqMan® SNP Genotyping Assays (Life Technologies, Carlsbad, CA, USA).

### 2.5. Statistical Analysis

Two-tailed Student's *t*-test and Fisher's exact test were used to compare the differences among SCARs and tolerant control demographic data (SPSS for Windows; IBM Corp., New York, USA). The risks for allopurinol-induced SCARs were calculated using the dominant model and univariate logistic regression analysis by SPSS software (IBM Corp., New York, USA). The Haldane modification of Woolf's formula was used among samples containing zero. The Bonferroni-corrected *P* value (Pc-value) was calculated by multiplying *P* value by 4 which is the number of multiple comparisons to account for the observed SNPs in this study, and Pc-value less than 0.05 was considered statistically significant. The individual sensitivity, specificity, positive predictive values (PPV), and negative predictive values (NPV) of the *HLA-B*^∗^*58 : 01* allele or the selected SNPs for the screening of allopurinol-induced SCARs were calculated.

The estimated linkage disequilibrium coefficients (*D*') and coefficient of correlation (*r*^2^) between the *HLA-B*^∗^*58 : 01* allele and the selected SNPs were calculated using the PLINK (V1.07) program.

## 3. Results

### 3.1. Comparisons of the Associations between the *HLA-B*^∗^*58 : 01* Allele and the Selected SNPs with Allopurinol-Induced SCARs

Ninety-six allopurinol-induced SCARs (i.e., 23 DRESS and 73 SJS/TEN patients) and 193 allopurinol-tolerant controls were recruited for the study. The demographic and clinical characteristics of the case and the control groups are shown in Table 1.

Of ninety-six allopurinol-induced SCARs patients, 90 patients (93.75%) carried the *HLA-B*^∗^*58 : 01* allele including 21/23 (91.30%) patients in the DRESS group and 69/73 (94.52%) patients in the SJS/TEN group (Table 2). Compared with the SCARs group, only 23 of 193 (11.92%) patients in the tolerant control group carried this allele (Table 2). Results from the univariate analysis show that the *HLA-B*^∗^*58 : 01* allele was strongly associated with allopurinol-induced SCARs with an OR of 110.87 (95% CI = 43.57–282.15, Pc-value = 2.05 × 10^−22^) (Table 2). The risk of allopurinol-induced DRESS was 77.61-fold (95% CI = 17.07–352.85, Pc-value = 7.12 × 10^−8^) in the patients who carried the *HLA-B*^∗^*58 : 01* allele compared with those did not carry this allele. Similar to that observed in SJS/TEN, the risk of allopurinol-induced SJS/TEN in the *HLA-B*^∗^*58 : 01* allele carriers was 127.50-fold (95% CI = 42.53–382.27, Pc-value = 1.99 × 10^−17^) (Table 2).

Among the three selected SNPs in chromosome 6, the rs2734583 in the *BAT1* gene showed the strongest association with allopurinol-induced DRESS with an OR of 64.56 (95% CI = 14.31–291.16, Pc-value = 2.35 × 10^−7^) followed by the rs3099844 in the *HCP5* gene (OR = 59.38, 95% CI = 13.21–266.97, Pc-value = 4.04 × 10^−7^) and the rs9263726 in the *PSORS1C1* gene (OR = 22.21, 95% CI = 7.10–69.46, Pc-value = 3.91 × 10^−7^) (Table 2). In contrast, the strongest association between allopurinol-induced SJS/TEN was observed with rs9263726 (OR = 63.60, 95% CI = 23.85–169.56, Pc-value = 4.22 × 10^−16^). When considering both DRESS and SJS/TEN as SCARs, these three SNPs were significantly associated with SCARs induced by allopurinol with the strength of association ranging from 45 to 60 (Table 2). All of these SNPs in the study populations were followed the Hardy–Weinberg equilibrium.

The sensitivity, specificity, NPV, and PPV of the *HLA-B*^∗^*58 : 01* allele compared with the three selected SNPs in chromosome 6 for the prediction of both DRESS and SJS/TEN caused by allopurinol are shown in Table 3. These parameters of the *HLA-B*^∗^*58 : 01* allele for the prediction of DRESS or SJS/TEN were the highest. The sensitivity, specificity, PPV, and NPV of these three SNPs for the prediction of SJS/TEN as well as SCARs were quite similar (Table 3). It should be noted that these values of the rs9263726 for the prediction of DRESS were relatively lower than those of the other two SNPs (Table 3).

Concerning the haplotypes of these three selected SNPs (rs9263726-rs2734583-rs3099844), 69.57% (16/23) of the patients in the DRESS group and 83.56% (61/73) of the patients in the SJS/TEN group carried the GA-TC-CA haplotype compared with 12.44% (24/193) in the controls (Table 2). About 80.83% (156/193) of the control patients carried the GG-TT-CC haplotype. A small number of patients in the case and the control groups carried other haplotypes (data not shown). Compared with the GG-TT-CC haplotype, the risk of allopurinol-induced DRESS in the patients who carried the CA-TC-GA haplotype was about 52.00-fold (95% CI = 11.24–240.51, Pc = 1.17 × 10^−6^) whereas that of allopurinol-induced SJS/TEN was about 99.13-fold (95% CI = 33.03–297.52, Pc = 9.90 × 10^−16^) (Table 2). Sensitivity, specificity, PPV, and NPV of the CA-TC-CA haplotype screening for the prediction of DRESS and SJS/TEN are shown in Table 3.

### 3.2. Concordance between the *HLA-B*^∗^*58 : 01* Allele and the Selected SNPs in Chromosome 6

Results from linkage disequilibrium (LD) analysis in the study population (combining data from the case group and the control group, *n* = 289) showed that each SNP showed strong linkage disequilibrium with the *HLA-B*^∗^*58 : 01* allele with *D*' values of more than 0.90 and *r*^2^ values of more than 0.8 (Table 4). Subgroup analysis of LD in the DRESS group revealed that these three SNPs were a complete LD with the *HLA-B*^∗^*58 : 01* allele (*D*' = 1.0) but perfect LD with an *r*^2^ of 1.0 was found only for the rs2734583 and rs3099844 but not for rs9263726 (*r*^2^ = 0.4524) (Table 4). For the SJS/TEN and the SCARs groups, these three SNPs were complete LDs with the *HLA-B*^∗^*58 : 01* allele (D' = 1.0) but the *r*^2^ values were less than 0.8 (Table 4).

## 4. Discussion

Allopurinol is an effective and cheap drug for the treatment of gout; however, allopurinol-induced SCARs may be life-threatening adverse drug reactions. Therefore, prediction for patients who may be at risk of these reactions is necessary to increase the safety of the drug. In line with previous reports, the results from this study clearly show that the *HLA-B*^∗^*58 : 01* allele was strongly associated with both phenotypes of SCARs including DRESS and SJS/TEN caused by allopurinol. High sensitivity and high specificity of the *HLA-B*^∗^*58 : 01* allele for the prediction of these life-threatening reactions were observed (93.75% and 88.08%) with the PPV and NPV of 79.65% and 96.59%. Compared with the *HLA-B*^∗^*58 : 01* allele, the selected SNPs in chromosome 6 showed relatively low sensitivity and specificity as well as low PPV and NPV for the prediction of allopurinol-induced SCARs. In contrast to previous reports in a Japanese population, the rs9263726, rs2734583, and rs3099844 SNPs were not complete and perfect LDs with the *HLA-B*^∗^*58 : 01* allele in a Thai population.

Results from univariate analysis revealed that the *HLA-B*^∗^*58 : 01* allele was strongly associated with both DRESS and SJS/TEN caused by allopurinol (Table 2). The OR of the *HLA*^∗^*58 : 01* allele for the SJS/TEN was about 1.6-fold higher than that of DRESS. The overall OR of this *HLA* allele for the prediction of both phenotypes of allopurinol-induced SCARs was 110.87 (95% CI = 43.57–282.15, Pc = 2.05 × 10^−22^). It is noteworthy that 86 patients of the SJS/TEN and 182 patients in the control groups are the same patients as reported in the previous study [4]. A lower OR between the *HLA-B*^∗^*58 : 01* and allopurinol-induced SCARs observed in the present study and a previous study was due to the absence of the *HLA-B*^∗^*58 : 01* allele in some of the SCARs patients that were currently recruited for the present study. Due to the high sensitivity, high specificity, high PPV, and high NPV observed in the present study and in other previous studies [5, 7, 15, 25], the *HLA-B*^∗^*58 : 01* allele screening has been proposed as a valid genetic marker for screening patients who may be at a high risk of allopurinol-induced SCARs. To date, guidelines and recommendations for *HLA-B*^∗^*58 : 01* allele screening prior to allopurinol administration particularly in certain ethnicities with a high frequency of *HLA-B*^∗^*58 : 01* allele carriers such as Han Chinese, Thai, and Korean populations have been released [17, 26]. Moreover, data from several countries including Thailand and Taiwan suggest that the *HLA-B*^∗^*58 : 01* allele screening is a cost-effective intervention for preventing allopurinol-induced SCARs [27, 28].

A recent study using a genome-wide association study in a Japanese population (including 14 allopurinol-related SJS/TEN patients and 991 ethnically matched controls) has discovered a set of SNPs in chromosome 6, particularly rs9263726 in the *PSORS1C1* gene, rs2734583 in the *BAT1* gene, and rs3094011 in the *HCP5* gene that were closely linked with the *HLA-B*^∗^*58 : 01* allele [13]. Moreover, results from a Chinese population revealed that among the three SNPs including rs9263726, rs2734583, and rs309984, the rs9263726 showed the highest degree of association with allopurinol-induced SJS/TEN (OR = 108.8, 95% CI = 13.9–850.5, Pc-value = 1.1 × 10^−7^) which was the same value as that of the *HLA-B*^∗^*58 : 01* allele [19]. Different to a report of a Chinese population, the OR values of the rs9263726 for the prediction of allopurinol-induced SCARs both DRESS and SJS/TEN in a Thai population were 2- to 3.5-fold lower than that of the *HLA-B*^∗^*58 : 01* allele (Table 2). Sensitivity, specificity, PPV, and NPV of the rs9263726 for the prediction of allopurinol-induced SCARs in a Thai population were relatively lower than those of the *HLA-B*^∗^*58 : 01* allele (Table 3).

The rs9263726 in the *PSORS1C1* gene has been reported to be complete LD (*D*' = 1) and perfect LDs (*r*^2^ = 1) with the *HLA-B*^∗^* 58 : 01* allele in a Japanese population (*n* = 206) [13]. Similarly, a recent study in 120 Chinese from the Eastern region of China (*n* = 120) showed a complete LD between the *HLA-B*^∗^*58 : 01* allele and rs9263726 (*D*' = 1) but their LD was not perfect (*r*^2^ = 0.92) [19]. In contrast, it has been reported in an Australian admixture population that the rs9263726 was not linked with the *HLA-B*^∗^*58 : 01* allele (*D*' = 0.059; *r*^2^ = 0.001) suggesting that these two alleles within nearby genes are inherited independently from each other in an Australian admixture population [29]. It should be noted that results from the present study showed that although the rs9263726 was in complete LD (*D*' = 1) with the *HLA-B*^∗^*58 : 01* allele in DRESS, SJS/TEN, SCARs, and the tolerant control groups but this SNP appeared to be not perfect LD with the *HLA-B*^∗^*58 : 01* because the *r*^2^ values were less than 1 (range from 0.4524 to 0.7884, Table 4). The lowest *r*^2^ values of this SNP were found with the allopurinol-induced DRESS. These results suggest that the rs9263726 may not be a good surrogate marker or good tag SNP of the *HLA-B*^∗^*58 : 01* allele for screening patients who are at risk of SCARs, particularly DRESS induced by allopurinol.

In comparison with the *HLA-B*^∗^*58 : 01* allele, it was found that the strength of association between allopurinol-induced DRESS and allopurinol-induced SJS/TEN and the rs2734583 or the rs3099844 were about 1.3–2.39-fold lower (Table 2). The sensitivity, specificity, PPV, and NPV of these two SNPs were comparable to those of the *HLA-B*^∗^*58 : 01* allele (Table 3). Although these two SNPs were complete (*D*' = 1) and perfect LDs (*r*^2^ = 1) with the *HLA-B*^∗^*58 : 01* allele in the allopurinol-induced DRESS, they were not perfect LD (*r*^2^ of 0.5466–0.8318) with the *HLA-B*^∗^*58 : 01* allele in SJS/TEN and tolerant control groups (Table 4). These results suggest that neither the rs2734583 nor the rs3099844 is a good surrogate marker of the *HLA-B*^∗^*58 : 01* allele for screening Thai patients who are at risk of SCARs. The rs2734583 and the rs3099844 appeared to be complete and perfect LDs in the SCARs group but not perfect LDs in the control group (data not shown). Detecting the haplotypes of these three SNPs was not significantly increased for the sensitivity, specificity, PPV, and NPV for the prediction of allopurinol-induced SCARs than single SNP detection (Table 3).

To date, the definite role of these SNPs in the pathogenesis of drug-induced SCARs is still unknown. The *PSORS1C1* gene encodes a psoriasis susceptibility 1 candidate gene 1 protein. Although the function of this protein is not clear, the *PSORS1C1* gene polymorphism has been reported to be associated with the susceptibility to psoriasis, hyper proliferative skin disorder [30, 31], and rheumatoid arthritis [32] whereas the *BAT1* gene encodes a protein that downregulated inflammatory cytokine production in splicing and RNA export mechanism such as tumor necrosis factor (TNF), interleukin-1, and interleukin-6 [33]. The polymorphism of the *BAT1* gene has been reported to be associated with rheumatoid arthritis [34]. The *HCP5* gene encodes a human endogenous retroviral element that sequences homology to retroviral *pol* genes. The *HCP5* was expressed in lymphocytes and suggested to control retrovirus proliferation via antisense mechanism [35]. Of interest, *HCP5* genetic polymorphism has previously been reported to be associated with nevirapine-induced SJS/TEN [36] and abacavir hypersensitivity [37]. It should be noted that the three SNPs investigated in the present study are located in chromosome 6p21.3 which is the same region as the MHC molecule well recognized as a key element for the pathogenesis of several drug-induced SCARs [38]. It is likely that the strong association between these three SNPs and allopurinol-induced SCARs may be due to linkage disequilibrium with the *HLA-B*^∗^*58 : 01* allele.

In summary, the three selected SNPs, rs926372, rs2734583, and rs3099844, were significantly associated with DRESS and SJS/TEN caused by allopurinol but the degree of associations was lower than that of the *HLA-B*^∗^*58 : 01* allele. The sensitivity, specificity, PPV, and NPV of these SNPs were comparable to those of the *HLA-B*^∗^*58 : 01* allele; however, these SNPs were not perfect LDs with the *HLA-B*^∗^*58* : 01 allele. Although the detection of the single SNP is more simple and less expensive compared with the detection of such polymorphic gene like the *HLA-B*^∗^*58 : 01* allele, results obtained from screening for the risk of allopurinol-induced SCARs using these SNPs as surrogate makers of the *HLA-B*^∗^*58 : 01* allele need to be carefully interpreted.

## Figures and Tables

**Table 1:** Demographic and clinical characteristics of allopurinol-induced SCARs and tolerant control patients.

Characteristic data		DRESS (*n* = 23)	SJS/TEN (*n* = 73)	SCARs (*n* = 96)	Control (*n* = 193)
Age (year)	Mean (SD)	65 (14)	65 (12)	65 (12)	64 (12)
	Median (range)	68 (28–82)	68 (38–84)	68 (28–84)	66 (29–91)
Gender	Female; *n* (%)	10 (43.48)	40 (54.79)^∗∗∗∗∗^	50 (52.08)^∗∗∗∗∗^	49 (25.39)
Onset of SCARs (day)	Mean (SD)	29 (13)	19 (11)	21 (13)	—
	Median (range)	30 (10–60)^∗∗∗∗∗^	18 (2–60)^∗∗∗∗∗^	20 (2–60)^∗∗∗∗∗^	—
Allopurinol dose (mg)	Mean (SD)	171 (78)	182 (106)	179 (99)	194 (90)
	Median (range)	200 (100–300)	100 (50–600)	100 (50–600)	200 (100–600)
Indication of allopurinol	Hyperuricemia; *n* (%)	11 (47.83)^∗∗∗∗∗^	13 (17.81)^∗∗∗∗∗^	24 (25.00)^∗∗∗∗∗^	3 (1.55)
	Gouty arthritis; *n* (%)	12 (52.17)	60 (82.19)	72 (75.00)	190 (98.45)
*Baseline kidney function*
Blood urine nitrogen (mg/dL)	*n*	*14*	*26*	*40*	*129*
	Mean (SD)	29.79 (21.14)	24.85 (20.09)	26.58 (20.33)	18.63 (10.40)
	Median (range)	21.35 (11.00–80.00)	18.50 (10.00–105.00)	21.00 (10.00–105.00)^∗^	16.20 (3.00–70.00)
Serum creatinine (mg/dL)	*n*	*19*	*65*	*84*	*174*
	Mean (SD)	2.20 (2.20)	1.54 (1.05)	1.69 (1.40)	1.47 (1.46)
	Median (range)	1.40 (0.90–9.60)	1.30 (0.50–8.82)	1.30 (0.50–9.60)	1.20 (0.70–14.80)
Estimated glomerular filtration rate (eGFR) (mL/min/1.73 m^2^)^a^	*n*	*19*	*65*	*84*	*174*
	Mean (SD)	44.05 (21.99)	48.61 (21.95)	47.58 (21.91)	58.01 (20.41)
	Median (range)	46.00 (6.54–88.61)^∗∗^	45.27 (4.62–127.17)^∗∗^	45.65 (4.62–127.17)^∗∗∗^	57.45 (3.12–112.49)
	eGFR < 30.00; *n* (%)	4 (17.39)	14 (19.18)^∗∗^	18 (21.43)^∗∗^	13 (7.47)
	30.00 ≤ eGFR < 60.00; *n* (%)	11 (47.83)	37 (50.68)^∗^	48 (57.14)	78 (44.83)
	eGFR ≥ 60.00; *n* (%)	4 (17.39)^∗^	14 (19.18)^∗∗∗^	18 (21.43)^∗∗∗∗^	83 (47.70)

^a^eGFR: estimated glomerular filtration rate (expressed as mL/min/1.73 m^2^) calculated by Modification of Diet in Renal the Disease (MDRD) study equation [39]. Indicated significant difference between case and tolerant control. ^∗^*P* value < 0.05, ^∗∗^*P* value < 0.01, ^∗∗∗^*P* value < 0.001, ^∗∗∗∗^*P* value < 0.0001, and ^∗∗∗∗∗^*P* value < 0.00001.

**Table 2:** Univariate analysis of the association between *HLA-B*^∗^*58 : 01* allele and SNPs with allopurinol-induced SCARs.

Allele/SNPs		Control (*n* = 193)	DRESS (*n* = 23)		SJS/TEN (*n* = 73)		SCARs (*n* = 96)	
		*n* (%)	*n* (%)	OR [95% CI] Pc-value	*n* (%)	OR [95% CI] Pc-value	*n* (%)	OR [95% CI] Pc-value
*HLA-B* ^∗^ *58 : 01*	Negative	170 (88.08)	2 (8.70)	Reference	4 (5.48)	Reference	6 (6.25)	Reference
	Positive	23 (11.92)	21 (91.30)	77.61 [17.07–352.85] 7.12 × 10^−8^	69 (94.52)	127.50 [42.53–382.27] 1.99 × 10^−17^	90 (93.75)	110.87 [43.57–282.15] 2.05 × 10^−22^

rs9263726 (G/A)	Negative (GG)	159 (82.38)	4 (17.39)	Reference	5 (6.85)	Reference	9 (9.38)	Reference
	Positive (GA/AA)	34 (17.62)	19 (82.61)	22.21 [7.10–69.46] 3.91 × 10^−7^	68 (93.15)	63.60 [23.85–169.56] 4.22 × 10^−16^	87 (90.63)	45.21 [20.73–98.60] 3.92 × 10^−21^

rs2734583 (T/C)	Negative (TT)	166 (86.01)	2 (8.70)	Reference	7 (9.59)	Reference	9 (9.37)	Reference
	Positive (TC/CC)	27 (13.99)	21 (91.30)	64.56 [14.31–291.16] 2.35 × 10^−7^	66 (90.41)	57.97 [24.07–139.60] 5.51 × 10^−19^	87 (90.63)	59.43 [26.76–131.97] 4.24 × 10^−23^

rs3099844 (C/A)	Negative (CC)	164 (84.97)	2 (8.70)	Reference	7 (9.59)	Reference	9 (9.38)	Reference
	Positive (CA/AA)	29 (15.03)	21 (91.30)	59.38 [13.21–266.97] 4.04 × 10^−7^	66 (90.41)	53.32 [22.26–127.71] 1.82 × 10^−18^	87 (90.63)	54.67 [22.77–120.66] 1.58 × 10^−22^

SNPs haplotypes (rs9263726-rs2734583-rs3099844)	GG-TT-CC	156 (80.83)	2 (8.70)	Reference	4 (5.48)	Reference	6 (6.25)	Reference
	GA-TC-CA	24 (12.44)	16 (69.57)	52.00 [11.24–240.51] 1.17 × 10^−6^	61 (83.56)	99.13 [33.03–297.52] 9.90 × 10^−16^	77 (80.21)	83.42 [32.74–212.55] 7.43 × 10^−20^

Pc-value: Bonferroni-corrected *P* value, calculated by multiplying *P* value by 4 which is the number of detected SNPs.

**Table 3:** Properties of proposed genetic screening tests for prediction of allopurinol-induced SCARs in a Thai population.

Allele/SNPs	Sensitivity (%)			Specificity (%)			PPV (%)			NPV (%)		
	DRESS	SJS/TEN	SCARs	DRESS	SJS/TEN	SCARs	DRESS	SJS/TEN	SCARs	DRESS	SJS/TEN	SCARs
*HLA-B* ^∗^ *58 : 01*	91.30	94.52	93.75	88.08	88.08	88.08	47.73	75.00	79.65	98.84	97.70	96.59
rs9263726 (G/A)	82.61	93.15	90.63	82.38	82.38	82.38	35.85	66.67	71.90	97.55	96.95	94.64
rs2734583 (T/C)	91.30	90.41	90.63	86.01	86.01	86.01	43.75	70.97	76.32	98.81	95.95	94.86
rs3099844 (C/A)	91.30	90.41	90.63	84.97	84.97	84.97	42.00	69.47	75.00	98.80	95.91	94.80
SNPs haplotypes (rs9263726-rs2734583-rs3099844)												
GA-TC-CA	69.57	83.56	80.21	87.56	87.56	87.56	40.00	71.76	76.24	96.02	93.37	89.89

Data presented as percentage. PPV: positive predictive value; NPV: negative predictive value.

**Table 4:** Concordance between selected SNPs and *HLA-B^∗^58 : 01* allele in allopurinol-induced SCARs.

SNP	Disequilibrium coefficient (*D*')				Coefficient of correlation (*r*^2^)			
	DRESS (*n* = 23)	SJS/TEN (*n* = 73)	SCARs (*n* = 96)	Control (*n* = 193)	DRESS (*n* = 23)	SJS/TEN (*n* = 73)	SCARs (*n* = 96)	Control (*n* = 193)
rs9263726	1.0000	1.0000	1.0000	1.0000	0.4524	0.7884	0.6444	0.6327
rs2734583	1.0000	1.0000	1.0000	1.0000	1.0000	0.5466	0.6444	0.8318
rs3099844	1.0000	1.0000	1.0000	1.0000	1.0000	0.5466	0.6444	0.7651
